# Isolated Ro52 Antibodies as Immunological Marker of a Mild Phenotype of Undifferentiated Connective Tissue Diseases 

**DOI:** 10.1155/2017/3076017

**Published:** 2017-01-22

**Authors:** Ana Alonso-Larruga, Sagrario Bustabad, José Antonio Navarro-Gonzálvez, Beatriz Rodríguez-Lozano, Andrés Franco, Yvelise Barrios

**Affiliations:** ^1^Biochemistry Unit, Central Laboratory, Complejo Hospitalario Universitario de Canarias, Tenerife, Spain; ^2^Immunology Unit, Central Laboratory, Complejo Hospitalario Universitario de Canarias, Tenerife, Spain; ^3^Rheumatology Department, Complejo Hospitalario Universitario de Canarias, Tenerife, Spain

## Abstract

The term undifferentiated connective tissue disease (UCTD) is used to describe undiagnosed patients that do not fulfill classification criteria for definite connective tissue disease (Systemic Lupus, Systemic Sclerosis, Sjögren Syndrome, and Dermatomyositis/Polymyositis). It is important to find serological markers as predictors of the evolution or severity of these diseases. The objective of this retrospective study was to investigate if there was a milder subgroup of UCTD with a special clinical profile consisting only in the presence of anti-Ro52 autoantibodies. Immunological and clinical records of 62 patients attending the hospital during 30 months were studied. Results showed a target population formed by mostly women, aged between 40 and 80 years at the moment of the study, with a registered age of onset between 40 and 60 years. Speckled pattern was the most frequent pattern found by indirect immunofluorescence. Given the obtained results and keeping in mind possible limitations because of sample size, isolated positive anti-Ro52 autoantibodies seem to lead to a benign effect in terms of evolution of the disease. As a future objective, the follow-up of these patients should be necessary to investigate new clinical symptoms, serological markers, or development of a definite connective tissue disease over time.

## 1. Introduction

Undifferentiated connective tissue disease (UCTD) refers to unclassifiable systemic autoimmune diseases which share clinical and serological manifestations with definitive connective tissue diseases (CTDs) such as Systemic Lupus Erythematosus (SLE), Systemic Sclerosis (SSc), Sjögren Syndrome (SS), Dermatomyositis/Polymyositis (DM/PM), Mixed Connective Tissue diseases (MCTD), and Rheumatoid Arthritis (RA) but not fulfilling any of the existing classification criteria [1, 2].

These disorders implicate disturbances of the immunological system with underlying inflammatory tissue injury. Although the trigger mechanisms remain unknown, there are some already established clinical and serological markers associated with these diseases [3]. The most common symptoms found in systemic rheumatic diseases are arthralgias (37–80%), arthritis (14–70%), Raynaud's syndrome (45–60%), leukopenia (11–42%), and other cytopenias, xerostomia (7–40%), xerophthalmia (8–36%), nonspecific rash, and oral aphthosis [4]. Moreover, there are several serological markers that can be found, such as ANA (90%), anti-Ro/SSA (8–30%), anti-RNP (10–30%), anti-dsDNA, or anti-phospholipid antibodies (Table 1). 80% of patients present single autoantibody specificity [2].

It is important to investigate the possibility of having good serological markers that will help clinicians predict the evolution or severity of these diseases. In this study the presence and clinical significance of autoantibodies against Ro52 without any other relevant serological marker were investigated.

Ro/SSA is a RNA protein particle in which the protein carries the major part of the antigenicity. There are two molecular forms of the protein (60 and 52 kDa). Anti-Ro52 antibodies were described for the first time in 1988 in patients with Sjögren Syndrome [5]. Along the past few years, new clinical associations have been opening (Table 2) due to their ubiquitous localization and distribution in many tissues [6]. Anti-Ro52 antibodies can also appear in patients without systemic rheumatic diseases, for example, in viral infections and neoplastic processes [7]. Despite these associations, the clinical significance of anti-Ro52 antibodies remains controversial [8].

Having in mind all this, a retrospective study was performed to analyse the clinical profile of patients having anti-Ro52 in their serum and not any other relevant autoimmune biomarker in order to investigate the possible association between anti-Ro52 antibodies and a milder and more stable form of UCTD. The evolution of the symptoms was evaluated to establish a conclusion regarding its possible prognosis value.

## 2. Materials and Methods

### 2.1. Studied Population

A database was prepared with the results stored on OpenLab™ records of the Immunology Section of Central Laboratory of Hospital Universitario de Canarias during 30 consecutive months. These records were obtained from patients attending the hospital to be tested for autoimmunity, regardless any specific clinical entity. Single centre, retrospective study was performed based on the positive results of immunoblotting panels for the detection of anti-Ro52 antibodies. Clinical data was investigated in all patients, specially oriented to the presence of autoimmune diseases.

All samples followed the routine protocol for studying anti-Ro52 autoantibodies, including a first screening step of antinuclear antibodies testing by indirect immunofluorescence (IFI) followed by an antigen-specific multiplex immunoblotting study in case there was either a positive result or high clinical suspicion for Ro52 analysis.

### 2.2. Indirect Immunofluorescence (IFI)

Anti-nuclear antibodies were detected by indirect immunofluorescence technique using slides containing HEp2 cells as substrate (Kallestad, Bio-Rad) following manufacturer's instructions. Briefly, serum from the patient is diluted 1 : 80 and incubated at room temperature for 30 min on HEp2 slides.

After extensive washing, a secondary FITC-conjugated anti-human immunoglobulin conjugate is added and incubated for 30 min. After a second washing step, mounting media are placed and the slides are read using Fluorescence Microscopy. Pattern and titre of positive samples were assessed by two different trained technicians.

### 2.3. Immunoblotting

Serum samples were analysed with commercial ANA Profile strips (Euroimmun, Medical Laboratory Diagnostic, Germany), which include the following antigens: Sm, RNP, SS-A (60 kD), Ro52, SS-B, Scl-70, PM-Scl, Jo-1, CENP B, PCNA, dsDNA, Nucleosomes, Histones, Ribosomal P-Protein, and AMA M2. The Ro52 antigen immobilized in the membrane is a recombinant antigen that has been obtained from baculovirus expression system in insect cells. Diluted serum samples (1 : 100) are incubated with these membrane strips with different antigens. In case of a positive sample, specific antibodies are seen as an intense black band at the line of the expected antigen. The analysis is performed using EuroblotMaster™.

### 2.4. Statistical Methods

Statistical analysis was performed using SPSS statistics programme (IBM). Contingency tables were built to investigate differences between diagnosed and undiagnosed patients for each symptom in the long or short time of evolution groups. The comparison analysis was made with Chi-squared test or Fisher's exact test when the previous one was not applicable. Probability values of *p* < 0.05 were considered to be statistically significant.

## 3. Results

During 30 months, the analysed sample population consisted in 377 Ro52 positive results. From those, 83 patients were found Ro52 positive without any other autoantibody that could be detected in relevant systemic autoimmune diseases (Sm, RNP, SS-A (60 kD), SS-B, Scl-70, PM-Scl, Jo-1, CENP B, PCNA, dsDNA, Nucleosomes, Histones, Ribosomal P-Protein, AMA M2, and Citrulline Peptide). 20 more patients were excluded from the study either because it was not possible to find enough clinical information about them (*n* = 15) or because they had no rheumatologic related symptoms (*n* = 5). These 62 remaining Ro52 positive patients were the focus of this study.

### 3.1. Demographic Details of the Sample Population

Of the 62 remaining patients, 92% were women (*n* = 57) and 8% were men (*n* = 5). The sex ratio was 10 : 1.

The average age found for the total population of study was 57 (±13) years (57 for women and 57 for men). Mode was 66 years old and median 59 years old. The most frequent age range was from 61 to 70 years (*n* = 17; 27%), followed by the rest of age ranges: from 51 to 60 years (*n* = 14; 23%), from 41 to 50 years (*n* = 12; 19%), from 71 to 80 years (*n* = 8; 13%), from 31 to 40 years (*n* = 7; 11%), over 80 years (*n* = 3; 5%), and from 21 to 30 years (*n* = 1; 2%). No patients were found in the range of <20 years of age.

**Figure 1:**
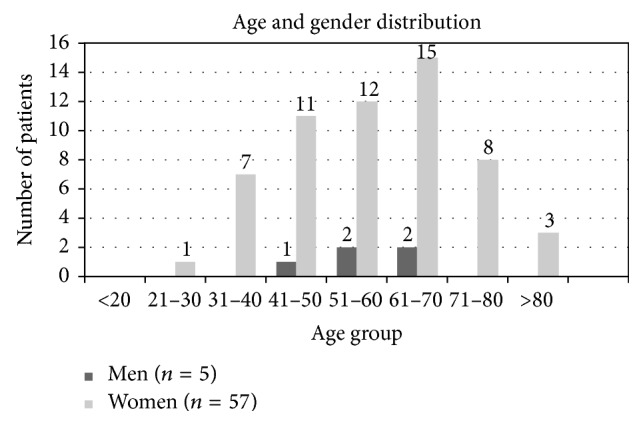
Age and gender distribution of the studied population (*n* = 62).

When combining gender and age data (Figure 1: age and gender distribution of the studied population), it was found that the population of patients was made mostly by women in the ages between 51 and 70. Positive results obtained from men were observed only in patients of more than 40 years of age.

### 3.2. Clinical Characterization of the Sample Population

The 62 patients were divided into two different groups:* Group 1*: patients with a definitive diagnosis *n* = 28 (45%) and* Group 2*: patients without a definitive diagnosis *n* = 34 (55%). A definitive diagnosis was either that for which the patient had been followed for a long period of time or that for which the antibody testing was requested. This group classification was based on information obtained after reviewing the medical records of each patient and collecting data related to clinical symptoms, time of evolution and possible or definitive diagnosis, and organising the data in a table used as a checklist with different items: arthralgia, arthritis, xerostomia, xerophthalmia, photosensitivity, nonspecific rash, alopecia, oral aphthosis, Raynaud's syndrome, haematological alterations, and systemic disease.

#### 3.2.1. Symptoms Distribution in Diagnosed and Undiagnosed Patients

**Figure 2:**
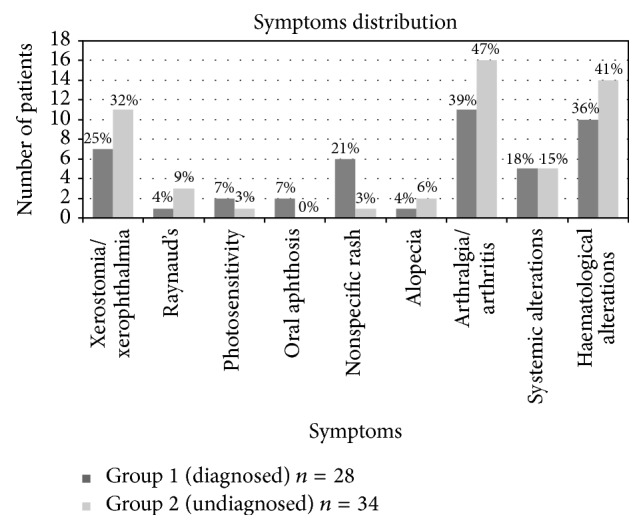
Distribution of symptoms in diagnosed patients and undiagnosed patients. Numbers indicate % of patients with each symptom among each group.

Data was collected from clinical history regarding which symptoms from the checklist were present in each case, depending on a positive response, a negative response, or clinical references found in medical records (Figure 2: distribution of symptoms in diagnosed patients (Group 1; *n* = 28) and undiagnosed patients (Group 2; *n* = 34); frequency in number of patients and % of patients with each symptom among each group).

*Group 1*:* Patients with Definitive Diagnosis (n* = 28*).* Based on ACR criteria for SLE and SS [9, 10], a distribution of the diagnosed diseases was obtained. 43% (*n* = 12) of patients were classified as having Systemic Lupus Erythematosus (SLE), 18% (*n* = 5) as Sjögren Syndrome (SS), 14% (*n* = 4) as Primary Biliar Cirrhosis (PBC), 7% (*n* = 2) as Lung Diseases, and 18% (*n* = 5) as other diagnoses. “Others” included Psoriatic Arthropathy (the only diagnosed patient with negative result for anti-ANA), Dermatomyositis, Atopic Dermatitis, and Graves Ophthalmopathy.

*Group 2*:* Patients without Definitive Diagnosis (n* = 34*).* This group of patients were the ones that did not fulfill enough criteria to be diagnosed as a CTD at the moment of the study. The results in Figure 2 showed that arthralgia and/or arthritis (*n* = 16), xerostomia (*n* = 10), xerophthalmia (*n* = 10), and haematological alterations (*n* = 14) were the most common symptoms to be found.

#### 3.2.2. Age of Onset in Diagnosed and Undiagnosed Patients

**Figure 3:**
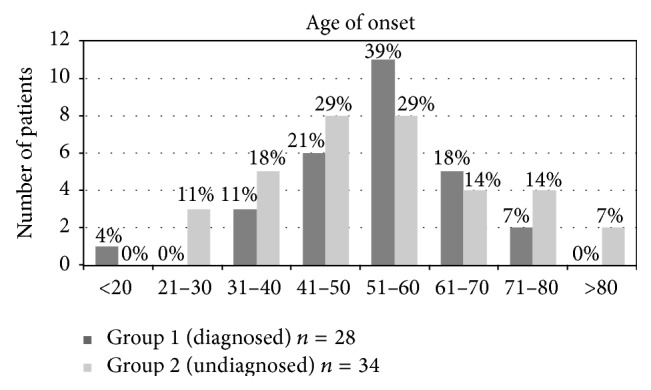
Distribution of age of onset in diagnosed and undiagnosed patients.

Data was collected from clinical history regarding the first time that symptoms appeared (Figure 3: distribution of age of onset in diagnosed and undiagnosed patients). 

*Group 1*:* Patients with Definitive Diagnosis (n* = 28*).* The most frequent age of onset range was from 51 to 60 years (*n* = 11; 39%), followed by the range 41 to 50 (*n* = 6; 21%).

*Group 2*:* Patients without Definitive Diagnosis (n* = 34*).* The most frequent age of onset ranges was from 51 to 60 (*n* = 8; 29%) and 41 to 50 (*n* = 8; 29%).

#### 3.2.3. Time of Evolution Distribution

*Group 1*:* Patients with Definitive Diagnosis (n* = 28*).* Patients were divided into two groups, representing the number of patients that had suffered the disease for less (≤3, *n* = 17) or more (>3, *n* = 11) than 3 years at the moment of the study. Data collected included average age of onset for each group, which was 56 and 47 years old, respectively. In these two groups, 2 of 17 of the patients with shorter time of evolution and 3 of 11 of the patients with longer time of evolution presented systemic alterations as kidney failure, neuronal alterations (epilepsy), or respiratory problems (serositis). That is, 18% of diagnosed patients presented systemic alterations.

*Group 2*:* Patients without Definitive Diagnosis (n* = 34*).* As in Group 1, a cut off of 3 years of evolution was used to divide the patients into short- or long-term symptoms evolution (≤3, *n* = 18; >3, *n* = 16). The average age of onset for each group was of 60 and 46 years, respectively. In this case, 2 of 18 of the patients with shorter time of evolution and 3 of 16 of the patients with longer time of evolution presented the same kind of systemic alterations. That is, 15% of undiagnosed patients presented systemic alterations.

#### 3.2.4. Time of Evolution Analysis

*Short-Term Evolution Patients (≤3 Years).* Statistically significant differences were not found between the groups regarding systemic alterations (Fisher's exact test), haematological alterations (Fisher's exact test), xerostomia, xerophthalmia, Raynaud's disease, oral aphthosis, photosensitivity, alopecia, rash, arthralgia, or arthritis.

*Long*-*Term Evolution Patients (>3 Years).* Statistically significant differences were found between groups regarding xerostomia (*p* = 0.005), xerophthalmia (*p* = 0.004), and arthritis (*p* = 0.027). A borderline *p* value (*p* ~ 0.005) was obtained regarding Raynaud's disease, oral aphthosis, and photosensitivity. No significant differences were found between the groups when analysing systemic alterations (Fisher's exact test), haematological alterations (Fisher's exact test), alopecia, rash, or arthralgia.

### 3.3. Immunological Characterization of the Sample Population

#### 3.3.1. Indirect Immunofluorescence

In the studied sample, 57 patients were ANA-positive (92%) and 5 were ANA-negative (8%) (routine Ag-specific protocol in these cases was performed after high suggestive clinical data was reflected in the patient's clinical records). Different fluorescence patterns were obtained in all positive samples: 31 speckled samples (50%), 15 homogeneous samples (24%), and 11 other patterns (18%).

*Group 1*:* Patients with Definitive Diagnosis (n* = 28*).* It was found that 16 samples had a speckled pattern (57%), 8 were homogeneous (29%), and 3 had other patterns (11%). 1 patient was ANA-negative (3%).

**Figure 4:**
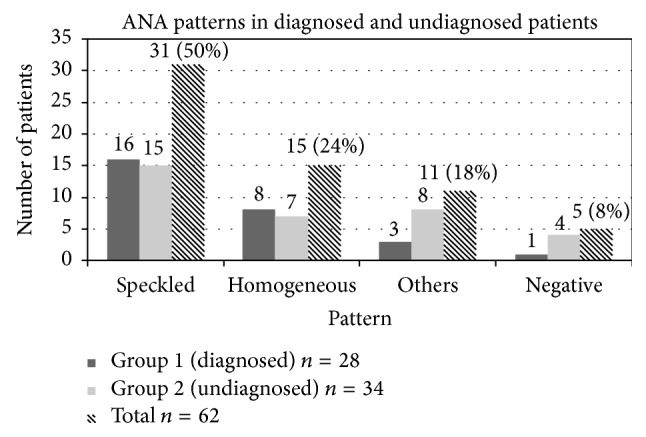
Distribution (in number of patients) of ANA patterns and ANA-negative results obtained in each group of patients.

*Group 2*:* Patients without Definitive Diagnosis (n* = 34*).* 15 patients (44%) were found with speckled pattern, 7 (21%) with homogeneous pattern, 8 (23%) with another kind of pattern, and 4 (12%) with negative ANA. (Figure 4: distribution of ANA patterns in number of patients and ANA-negative results obtained in each group of patients).

## 4. Discussion

Antibodies against Ro52 have been described in patients with a broad spectrum of autoimmune diseases, but usually coexisting with other several autoantibodies depending on the clinical association [7, 8]. However, the significance and clinical phenotype of isolated anti-Ro52 antibodies are not well known [11].

The major difference between the current series of anti-Ro52 serum and the ones reported by other groups is that these results are a consecutive blinded collection of the data obtained in a routine laboratory, rather than within a selected disease-biased population. For that reason, we decided to start collecting the medical records of those patients that were positive for isolated Ro52 autoantibodies. The protocol used to obtain ANA results includes a first screening IFI step followed by a second antigen-specific technique only in cases of positive ANA-IFI samples. Given the retrospective characteristic of the designed study, it is possible that some samples Ro52 positive but ANA-IFI negative were not detected (there are few cases in which the cytoplasmic coexpression of the antigen could affect these results). Indeed, in this cohort, a few ANA-IFI negative cases were included.

The comparison between the demographic characteristics (age and gender distribution) of the cohort of isolated Ro52 positive patients with those reported in the literature for global rheumatologic diseases shows that there is a certain concordance among them. According to already published studies, rheumatologic diseases are more frequent in women than in men [12]. SLE is mostly diagnosed for the first time between 10 and 50 years of age [13] whereas SS onset occurs in patients older than 40 [14]. In this line, isolated Ro52 patients are more frequently women (92%). Regarding the onset age, undiagnosed isolated Ro52 positive patients are older (mean onset age 53,6) if we compare them with the group of isolated Ro52+ with SLE (mean onset age of 49,2) and younger than isolated Ro52+SS patients in this series of patients (mean onset age of 61,8) (data not shown). Moreover, all the patients from the male group were older than 40 years old.

It has been published that 25% of rheumatic patients remain undiagnosed due to the lack of symptoms that prevents them to have a definite diagnosis of a known disease [3, 4, 15]. In our case, we had almost the same amount of diagnosed (45%) and undiagnosed (55%) patients, probably because of the peculiarities of the designed study including certain limitations (patients were followed by many practitioners of different areas of specialization and there was no standardization in follow-up or clinical registries). This result could also be influenced by the use of 2012 preliminary ACR criteria to classify the patients.

As expected, the majority of patients (21 out of 28) belonging to the “definitive diagnosis” group has a systemic autoimmune disease as the relevant diagnosis (SLE+SS+PBC: 75%), although a minority Ro52 autoantibodies can also be detected in some other clinical conditions (organ-specific autoimmune diseases included).

The majority of the total isolated Ro52 patients are in the short evolution (<3 years) period group (35 out of 62). When we analyse the time of evolution of the “definitive diagnosis” group, the tendency is that patients are in a short evolution period after clinical onset. However, there are 11 patients that have been followed for more than 3 years, and still Ro52 isolated antibody is the only antibody found in their serum sample. Again, there are more patients (18 versus 16) in the short evolution period (<3 years) on the “not definitive diagnosis” group.

On the other hand, it was found that only diagnosed patients with longer time of evolution (>3 years) have arthritis (*p* = 0.027), xerostomia (*p* = 0.005), and xerophthalmia (*p* = 0.004). Regarding oral aphthosis and photosensitivity, borderline results were obtained, so the analysis should be carried out in more patients in order to determine their statistical significance.

Although the small sample size limits the power of this study and more time of follow-up is needed, this paucity in the immune response (an isolated Ro52 autoantibody specificity in 27 patients for more than 3 years) could indicate that this group of patients may be expressing a milder form of these undifferentiated autoimmune diseases. To validate this, 12 months after the end point of our study, another review was performed, finding out that only 1 out of 35 undiagnosed patients suffered changes in symptoms or serological markers, giving the clinicians enough data to diagnose the patient with Sjögren Syndrome (data not shown). For that reason, we believe that this group of isolated Ro52 positive patients seem to be stable for longer periods, avoiding the epitope spreading effect which is frequently seen in other classical autoimmune diseases [16] with more aggressive immune response.

Regarding the IFI pattern of isolated Ro52 patients, there is no difference compared with the classical description [17]. As previously described, the majority of samples exhibited a speckled pattern, being the homogeneous pattern the second most frequent. Although fine specificity studies are out of the focus of this study, it may be interesting to investigate possible differences in the epitope that is recognized among isolated Ro52 positive patients compared with the ones recognized by SS autoantibodies [18], to see if this can influence the outcome of the immunological characteristic of this autoantibody in cases where no other antigens are relevant in the evolution of the disease.

## 5. Conclusions

Even though the study had limitations, especially regarding the gathering of the patients and the number of patients found, it is possible to obtain a major conclusion consisting in the description of a population of Ro52 isolated patients that has clinical and immunological characteristics that seems to be different from other Ro52 associated clinical entities.

As future directions, it will be necessary to do a close follow-up of our population of study to see if there is any change in the serological profile or symptoms and how many of them develop a CTD over the years, preparing a previously agreed checklist to standardize the information obtained by clinicians to elaborate a new and improved database.

## Figures and Tables

**Table 1:** Clinical and serological markers that predict the evolution to each definite connective tissue disease (CTD).

Disease	Predictive evolution factors	
	Clinical	Serological
Systemic Lupus Erythematosus (SLE)	Serositis Alopecia Photosensitivity Discoid rash	Anti-dsDNA Anti-Sm Antiphospholipid
Sjögren Syndrome (SS)	Raynaud Xerostomia	Anti-Ro Anti-La
Systemic Sclerosis (Scl)	Raynaud Sclerodactyly Esophageal dysfunction	ANA nucleolar pattern
Rheumatoid Arthritis (RA)	Polyarthritis	Rheumatoid factor

**Table 2:** Classical clinical associations of anti-Ro52 antibodies.

Disease	% patients
Sjögren Syndrome (SS)	59
Systemic Lupus Erythematosus (SLE)	32
Systemic Sclerosis (Scl)	21
Idiopathic Inflammatory Myopathies (IIM)	19
Rheumatoid Arthritis (RA)	15
